# Are “European” Scrotal Hernias Repairable With the Minimal Open Pre-Peritoneal Technique?

**DOI:** 10.3389/jaws.2025.13863

**Published:** 2025-02-20

**Authors:** Marc Soler, Jean Francois Gillion

**Affiliations:** ^1^ Clinique Saint Jean, Cagnes-sur-Mer, France; ^2^ Antony Private Hospital, Antony, France

**Keywords:** MOPP, scrotal hernia, open surgery, pre peritoneal, prosthesis

## Abstract

**Background:**

Minimally invasive open preperitoneal techniques are an alternative in groin hernia repair. Scrotal hernias (SH) are frequently difficult to repair laparoscopically, resulting in a significant conversion rate.

**Methods:**

The aim of this exploratory monocentric retrospective study, based on data prospectively collected in the “Club-Hernie” registry, was to assess the feasibility, effectiveness and safety of the MOPP technique in SH repair compared with non-SH repair.

**Results:**

All consecutive MOPP repairs performed from 11 September 2011 to 31 December 2022 were identified in which 2005 MOPP (126 SH and 1879 non-SH) met the inclusion criteria. The results were analysed “as treated” in 125 SH vs. 1879 non-SH. No statistically significant difference was observed between these two groups in terms of age, BMI, and ASA classification. Symptomatic hernias (84% vs. 73%; p < 0.001), and lateral hernias (87.80% vs. 62.81%; p < 0.0001) were more frequent in the SH group. The mean operating time was longer (58 min vs. 39 min; p < 0.0001) in the SH group. The SH procedures were performed under general anaesthesia with a laryngeal mask in 92% of cases. All postoperative complications, except one reoperation in the non-SH group, were classified as Clavien-Dindo Grade I/II. Superficial surgical site occurrences were more frequent in the SH group (14% vs. 3%; p < 0.0001). No peri-prosthetic infections were observed. The outpatient rate was 83% vs. 94% in the SH and non-SH groups, respectively. There were four rehospitalisations in the non-SH group and none in the SH group. The postoperative pain was low and similar in the two groups, except at M1, where the mean pain was lower in the SH group (p < 0.001). A total of 113 (90%) patients in the SH group vs. 1,553 (82%) in the non-SH group were followed for 1 year or more. The number of identified recurrences and reoperations was low and did not differ between the two groups studied. In total, 98% of patients in both groups assessed their surgery as excellent or good.

**Conclusion:**

This exploratory study shows that the MOPP technique is feasible and safe in scrotal hernia repair, with similar results to those observed in non-scrotal hernias. Our next step will be to compare MOPP with laparoscopic and Lichtenstein techniques in scrotal hernia repair.

## Introduction

The concept of minimally invasive open surgery for groin hernia repair dates back to approximately 20 years ago. It adopts the principle of utilising a preperitoneal prosthesis advocated over 60 years ago by Franz Ugahary, who pioneered the minimally invasive concept in groin hernia repair with his supra-inguinal grid-iron technique through a very small incision, thus requiring specific long and smooth retractors [1, 2]. A few years after the TIPP (transinguinal preperitoneal) technique was described [3, 4], using a minimally invasive inguinal route and a mesh equipped with a memory ring [5], which was inserted in the preperitoneal space after parietalisation of the spermatic cord [6, 7]. Another variant is the trans-rectus preperitoneal (TREPP) technique [8, 9], and the last variant is the minimally open pre peritoneal (MOPP) technique which is based on Ugahary’s principles (similar set of retractors) but with a deep inguinal ring [10–12]. In the majority of the published comparative studies, the results of the minimally invasive open preperitoneal techniques were found to be superior to those of the Lichtenstein technique, especially in reducing the incidence of chronic postoperative inguinal pain (CPIP) [13, 14]. Other studies show almost similar results between preperitoneal and laparoscopic methods [15, 16], except in the study by Reinhold et al. [17], which demonstrated a potential benefit in short-term quality of life and seroma formation with open posterior mesh placement compared to minimally invasive surgery (endoscopic, robotic) repair.

However, are we allowed to extrapolate these results to larger hernias (e.g., scrotal hernias), which are known to be more difficult to fix [18] Are they repairable with minimally invasive open inguinal techniques, especially the MOPP technique? A scrotal hernia is commonly defined as an inguinal hernia that, in the upright position, descends into and causes any distortion of the scrotum [18].

In the classification proposed by Tran et al. [18] the scrotal hernias are subdivided into S1 (upper third of the thigh), S2 (middle third of the thigh), S3 (lower third of the thigh/patella), and Sn (IR) in case of irreducibility. In high-income countries, where scrotal hernias type S2 or S3 [19] are very rare, recent guidelines [18] recommend an open mesh repair (e.g., Lichtenstein) or a totally extraperitoneal (TEP) laparoscopic repair for a large reducible scrotal hernia, while they recommend a trans-abdominal preperitoneal laparoscopic (TAPP) repair for an irreducible hernia. Due to the lack of published data, open repair other than Lichtenstein have not been considered in the key questions of these scrotal hernia repair guidelines. Thus, the main objective of the present exploratory monocentric prospective study was to investigate the feasibility, effectiveness and safety of the MOPP technique in the repair of S1 scrotal hernias (SH) compared to non-scrotal hernias (NSH).

## Methods

This retrospective cohort study was conducted according to the STROBE [20] statement, and the recommendations of the European Registry of Abdominal Wall Hernias working group [21].

### Study Design

We conducted a comparative study of data prospectively collected in the “Club-Hernie” database. All consecutive MOPP repairs performed by the same surgeon from 11 September 2011 to 31 December 2022 for primary groin hernias, either scrotal (SH) or non-scrotal (NSH) were included and compared. The exclusion criteria were as follows: Hernia repair in female patients, history of radical prostatectomy, vascular bypass, or pelvic irradiation; Recurrent hernia, emergent hernia, or pure femoral hernia (not combined with an inguinal hernia).

### Club Hernie Registry

The registry complies with the European General Data Protection Regulation (GDPR) [22]. The study’s registry-based design, which guarantees that all data are anonymous and de-identified, collected with a patient “non-opposition” agreement, complies with the national ethical standards of the French “Commission Nationale de l’Informatique et des Libertés” (CNIL) (registration number: 1993959v0).

### Studied Surgical Technique

The MOPP technique has already been published in scientific articles [10, 12], and book chapters [11]. Briefly, it consists of i) Dissecting the preperitoneal space through minimal inguinal access, smaller than that of TIPP, using long, thin and smooth specific blade dissectors and retractors, ii) Reintegrating the hernia sac into the abdominal cavity, iii) Inserting a preperitoneal flat mesh equipped with a memory ring through the deep inguinal ring, facilitating its deployment. The modifications to the MOPP technique required for treating scrotal hernias are as follows: The skin incision is to be enlarged from 25–40 mm to 40–60 mm. Priority is given to the recognition, dissection and sometimes resection of the sac before isolation of the spermatic cord, which is not spontaneously accessible. Recognition of the hernial sac is difficult as the elements of the cremaster cannot simply be pushed back inside as in the basic MOPP technique [12]. The presence of fibrous tissue around the sac and the cord elements also makes it difficult to identify them, along with the ilioinguinal nerve and the genital branch of the genito-femoral nerve. One solution is to search and gently dissect the sac from its distal part towards its cranial part, separating it from the tissues and vessels that are initially difficult to identify. The management of the cremasteric fibres is different than in other techniques. They must be cut rather than pushed inwards [12]. The fifrous bundles witch have accompanied the evolution of these old hernias mast also be cut to facilitate the access to the deep inguinal ring. Extra care is needed to identify the spermatic vessels, the ilio-inguinal nerve and the genital branch of the genito-femoral nerve. Resection of a damaged nerve is sometimes required [18]. The distal part of the sac, when adherent to the scrotal contents, must be transected and left wide open. The rare medial sacs that are large enough to develop in the scrotal area, are repaired in the same ways as others. When reducing the sac, as visual control of the epigastric vessels can be difficult, it is necessary to use retractors gradually without exerting strong pressure to avoid injury especially to the vein.

### Follow-Up, PROM Assessment and Late Complication Identification

CH members themselves register pre-, intra-, and 30-day postoperative data in the online database. Data entry is completed during the systematic clinical visit at month 1 (M1) scheduled with the operating surgeon. An optional clinical visit at month 3 (M3) is scheduled in case of any problems identified at M1. Subsequently, the dedicated Club-Hernie clinical research assistant (CRA), independent of the surgical teams, will manage the 1-2, and 5-year follow-up of the patients, following a formatted telephone PROM questionnaire, which has been used in our clinical studies since 1999 [23], during which the patients are systematically queried about rehospitalisation (in the same hospital or another one), reoperation and their causes, confirmed recurrence (reoperated, TDM/ultrasound, and/or surgeon visit), suspected recurrence (PINQ-Phone manoeuvre [24], localised bulging and/or local pain), late abscess, chronic sinus, mesh removal, and other late complications (e.g., bowel obstruction). After five unsuccessful attempts to contact the patient at various times and dates, they are deemed lost to follow-up. In the event of any deviation from the normal course, a visit to the surgeon’s office is strongly recommended. Additionally, some surgeons, like the first author, encourage their patients to attend systematic clinical visits, the results of which are recorded independently from those of the CRA, in surgeon dedicated tabs.

### Variables Used for the Present Study

Baseline variables extracted comprised: age, gender, body mass index (BMI), ASA classification, diabetes mellitus, hernia recurrence, smoking status, emergency surgery, synchronous repair of multiple defects, wound classification (clean, clean-contaminated, contaminated, dirty), type of hernia according to the European Hernia Society groin hernia classification simple and easy to remember [25] and the Tran H.M. et al. classification [18], surgical operative time, and length of stay. Intra-operative complications were defined as one or more of the following complications: peritoneal tear, bladder injury, bowel injury, orchidectomy, severe bleeding, or general complications that occurred during the procedure. Postoperative complications were clustered as follows: i. General complications including isolated or combined medical complications such as heart attack, thrombophlebitis with or without pulmonary embolism, compartment syndrome, neurological, arrhythmia, urinary retention, injection site inflammation within 30 days of surgery; ii. Surgical site infection (SSI) including all wound infections individualised into peri- (deep) or not peri-prosthetic (superficial) infected collections, and surgical site occurrence (SSO) including all peri- or non periprosthetic non-infected collections; iii. Organ space (surgical) complications including intraperitoneal bleeding, peritonitis, bowel obstruction, and immediate recurrence; In the case of concurrent complications, the Clavien-Dindo grading [26] was based on the worst complication. Postoperative pain was evaluated at D1, D2, D8, and D30 using a 0–10 VAS and compared with the 0–10 VAS preoperative status. Chronic postoperative inguinal pain (CPIP), defined as pain lasting more than 3 months, was evaluated during follow-up with 0–10 NRS, and 4 VRS scales (no pain, mild pain, moderate pain, severe pain) and compared with the preoperative status. Recurrences were clustered into reoperated recurrences, recurrences not reoperated but confirmed (CT scan, ultrasound, surgical clinical visit) and suspected recurrences.

### Outcomes of Interest

Feasibility, assessed by conversion rate, and intraoperative complicationsSafety, assessed by D30 and late complications Effectiveness, assessed by recurrence rate Patient self-evaluation, assessed with systematic pain evaluations, PROMs, and Q.O.L questionnaires.

### Descriptive Statistics

Discrete variables have been presented as absolute numbers and percentages. Continuous variables have been presented as mean +/− standard deviation (SD). Discrete variables have been compared using the Chi-square test or Fischer exact test, and continuous variables have been compared using the Student’s T-test.

## Results

### Flow Chart

**FIGURE 1:**
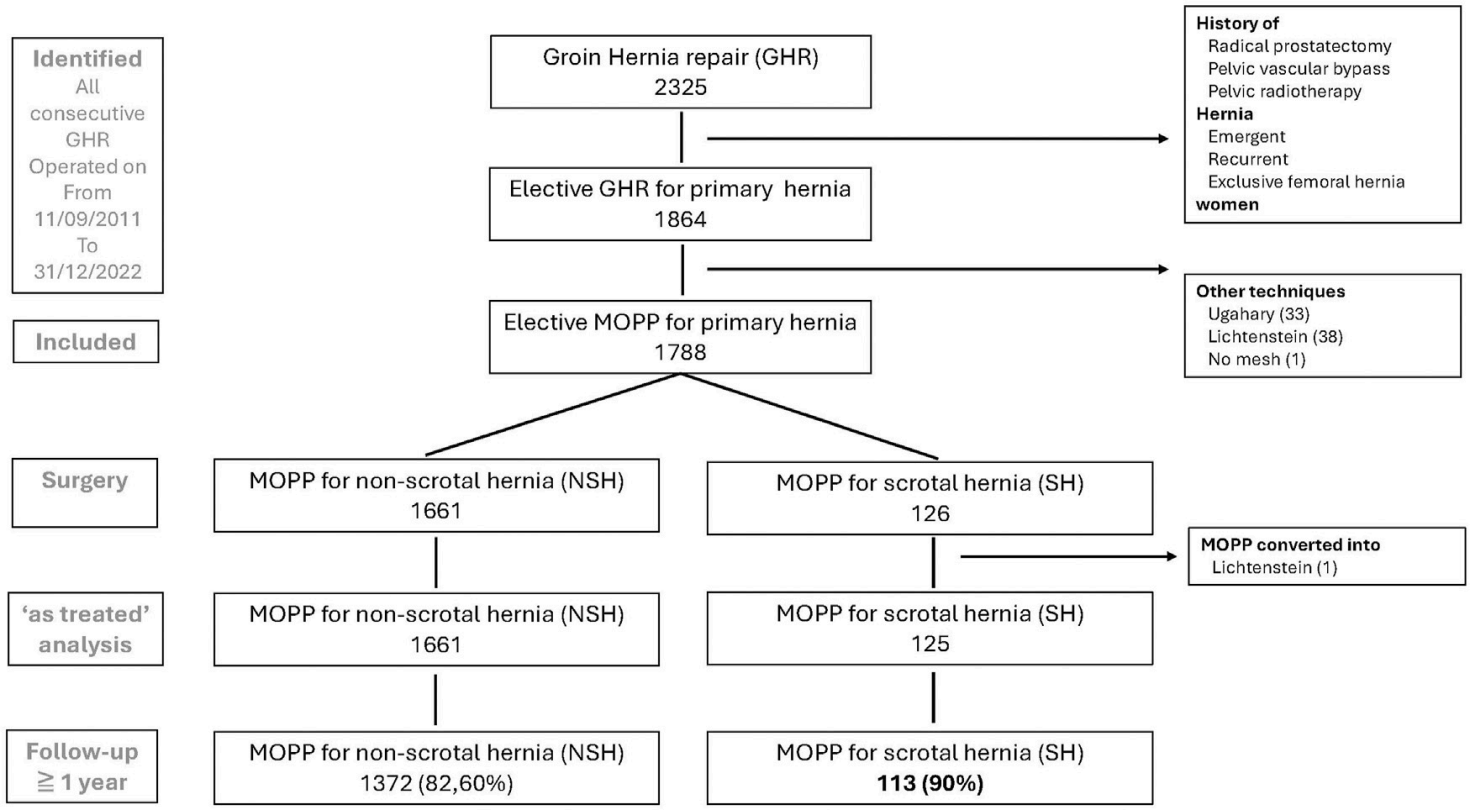
Flow chart.

From 11/09/2011 to 31/12/2022 a total of 2,325 groin hernias were operated on by the same operating surgeon, of which 2005 hernias, 126 scrotal (SH) and 1,661 non-scrotal hernias (NSH) matched the inclusion criteria (Figure 1). In one SH case, MOPP was converted to Lichtenstein due to dissection difficulties. Thus, in this series, the MOPP conversion rate for scrotal hernia repair was 0.79%. The results were further analysed “as treated” for 125 SH vs. 1661 non-SH subjects, and not in an “intend to treat” manner.

### Demographics, Pain Status and Q.O.L at Baseline

The two groups were similar in terms of age, BMI, comorbidities and ASA classification (Table 1). Patients with any preoperative pain or discomfort, especially VRS severe pain (28.22% vs. 16.67%) p < 0.01 or VAS 4–10 (49.45% vs. 35.50%; p < 0.05) were significantly more frequent in the SH patients. Their preoperative quality of life (Q.O.L) was significantly more impaired than that of the NSH group.

**TABLE 1:** Patients’ characteristics.

N (%) or mean +/− SD (range)	NSH	SH	*P*. value
MOPP repairs only Males	1,661	125	
Age (years)	69.08 ± 13.91	68.86 ± 18.1	P > 0.05
BMI (kgs/sqm)	24.62 ± 2.67	24.93 ± 4.3	P > 0.05
Diabetes mellitus	52 (3.13)	1 (0.80)	P > 0.05
Anticoagulant, antiplatelet	234 (14.08)	16 (12.80)	p > 0.05
Active smoker	324 (19.56)	26 (20.80)	p > 0.05
ASA classification			
Missing data	152 (9.15)	14 (11.2)	
ASA 1-2	1,460 (96.76)	106 (95.49)	
ASA 3-4	49 (3.14)	5 (4.50)	p > 0.05
Preoperative pain (0–10 VAS)			
Missing data^a^	492^a^	34*	
VAS 0–3	754 (64.50)	46 (50.55)	P < 0.05
VAS 4–10	415 (35.50)	45 (49.45)	
Preoperative pain (VRS)			
Missing data	6 (0.36)	1 (0.80)	
No pain	452 (27.31)	19 (15.32)	p = 0.01
Any pain	1,203 (72.68)	105 (84.67)	
Mild pain with uncommon pain	485 (29.30)	42 (33.87)	
Moderate	442 (26.70)	28 (22.58)	
Severe	276 (16.67)	35 (28.22)	P < 0.05
Preoperative PROM (Q.O.L)			
Missing data	9 (0.54)	1 (0.80)	
No preoperative symptom	447 (27.058)	19 (15.32)	P < 0.05
Preoperative symptoms	1,205 (72.94)	105 (84.68)	
Do not interfere with your daily life	479 (28.99)	29 (12.39)	
Allow to pursue the ongoing activity	195 (11.80)	26 (20.96)	
Cause a temporary interruption of your activity	174 (10.59)	13 (10.48)	
Prevent certain activities (impairment)	357 (21.61)	37 (29.63)	

VRS, Verbal Rating Scale; VAS, Visual Analogic Scale; NRS, Numeric Rating Scale Preoperative VAS.

^a^ Was introduced in the registry in 2015.

Percentages were calculated on not-blank values.

### Hernia Characteristics and Intraoperative Details

In almost 92% of both the NSH and SH groups, MOPP repairs were performed under general anaesthesia with a laryngeal mask without tracheal intubation (Table 2). Spinal anaesthesia was rare but significantly more frequent (0% vs. 0.48%; p < 0.0001) in the NSH group. The groin hernias treated were significantly different between the SH and NSH groups: Lateral inguinal hernias were more frequent (87.80% vs. 61.33%; p < 0.0001) in the SH group. Combined inguinal and femoral hernias were encountered in 1.58% vs. 0.81% of cases (p > 0.05). All SH hernias were S1 type according to the Tran H.M. classification [18]. Three types of preperitoneal mesh were successively used depending on their availability on the market during the study period. A large mesh (according to the manufacturer’s specifications) was used more frequently (83.20% vs. 65.31%; p < 0.0001) in the SH group. No mesh fixation was used in the scrotal group or in all but five cases (0.30%) in the NSH group. Intraoperative adverse events were very rare in each group and were not more frequent in the SH group. The operating time was longer (58 min vs. 39 min; p < 0.0001) in the SH group.

**TABLE 2:** Hernia characteristics/Intraoperative details.

N (%*)* or mean +/− SD (range)	NSH	SH	*P*. value
Cases	1,661	125	
Type of anesthesia			
Missing data	3 (0.18)	0 (0)	
General anesthesia intubation	52 (3.13)	6 (4.80)	p > 0.05
General anesthesia laryngeal mask	1,536 (92.64)	115 (92.00)	
Spinal	8 (0.48)	0 (0)	p < 0.0001
Local or regional block	62 (3.74)	4 (3.20)	p > 0.05
Altemeier			
Missing data	750 (45.15)	59 (47.20	p > 0.05
Clean	911 (100)	66 (100)	
Hernia EHS classification			
Missing data	3	2	
Lateral	1,017 (61.33)	108 (87.80)	p < 0.0001
L1	167 (16.12)	0 (0.00)	
L2	793 (77.97)	24 (22.22)	
L3	57 (5.60)	84 (77.77)	
Medial	687 (41.43)	15 (12.19)	P < 0.0001
M1	41 (5.96)	0 (0.00)	
M2	458 (66.66)	2 (13.33)	
M3	188 (27.36	13 (86.66)	
Lateral + medial	46 (2.77)	1 (0.81)	P > 0.05
Femoral only	0 (0.00)	0 (0.00)	p > 0.05
Femoral et lateral	12 (1.18)	2 (0.81)	p > 0.05
Femoral et medial	4 0.40)	0 (0.00)	p > 0.05
Mesh type			
Missing data	9 (0.54)	0	
Surgimesh™	745 (45.09)	45 (36.00)	P < 0.05
Polysoft™	54 (3.27)	7 (5.6)	
Onflex™	850 (51.45)	73 (58.40)	p > 0.05
Other	3 (0.18)	0	
Mesh size			
Missing data	12 (0.72)	0	
Large	1,077 (65.31)	104 (83.20)	p < 0.0001
Medium	572 (34.69)	21	
Mesh fixation			
Missing	5 (0.30)	0	
No	1,651 (99.70)	125 (100)	P > 0.05
Yes	5 (0.30)	0	
Intra operative adverse events			
Iliac vessels injury	0	0	
Bowel injury	0	0	
Bladder injury (sutured)	1 (missing data = 20) (0.06)	0	
Operating time			
Mean +/− SD (min)	39 (9.87)	58 (21)	p < 0.0001

Percentages were calculated on non-empty values.

### Day-30 Postoperative Outcomes

General (non-surgical) complications occurred rarely, with the same frequency (1.52% vs. 1.60%; p > 0.05), in each group. Surgical site occurrence (SSO), were more frequent (14.40% vs. 2.98%; p < 0.0001) in the SH group, consisting only of seromas (Table 3). One superficial (non-periprosthetic) surgical site infection occurred in the NSH group. Two organ-space complications, orchitis (N = 1) and deep haematomas (N = 1) occurred in the control group, and none in the SH group. No bowel obstruction, peritonitis, mesh removal occurred in the entire MOPP series. No reoperation or rehospitalisation were required in the SH group vs. one and four respectively in the NSH group. With the exception of one complication in the NSH group, all postoperative complications were benign, classified as Clavien I or II. Compared to the control group, the mean postoperative pain (VAS) in the SH group was (4.1. vs. 4.35; p > 0.05) at D1, (1.7 vs. 1.8; p > 0.05) at D8 and (0.40 vs. 0.71; p < 0.0001) at D30; the difference was statistically significant only at D30, in favour of SH.

**TABLE 3:** Day-30 postoperative outcomes.

N (%) or mean +/− SD	NSH	SH	*P*. value
Cases	1,661	125	
Postoperative complications			
Missing data	20 (1.20)	0 (0.00)	
General	25^a^ (1.52)	2^b^ (1.60)	P > 0.05
SSO			
SSO non-SSI	49^c^ (2.98)	18^d^ (14.40)	p < 0.0001
Non-periprosthetic SSI	0	0	
Periprosthetic SSI	0	0	
Surgical non SSO	2^e^ ^,^ ^f^	0	
Reoperation	1^e^	0	
Mesh removal	0	0	
Rehospitalization	4^g^	0	
Clavien classification			
Missing data	25	0	
Patient without complication	1,606	105	
Patient with any complication	30	20	P < 0.001
Grade I/II	29 (1.77)	20 (16.00)	
Grade III b	1 (0.06)	0	
Grade IV	0	0	
Grade V	0	0	
Postoperative pain (0–10 VAS)			
D1: mean (SD); missing	4.35 (2.12); 44	4.1 (2.01); 4	p > 0.05
D8: mean (SD); missing	1.8 (1.77); 45	1.7 (1.9); 4	p > 0.05
D30: mean (SD); missing	0.71 (1.41); 191	0.40 (0.99); 20	p < 0.0001
Missing data	9	0	
Outpatients	1,570 (95.04)	104 (83.20)	<0.0001
Inpatients	82 (4.96)	21 (16.80)	

Percentages were calculated on non-empty values.

SSO, Surgical site occurrence; including SSI, Surgical site infection.

Clavien Dindo classification (REF.): In case of combined complications the CDC grading (per patient) was calculated on the worse complication VAS: Visual analogic scale; D1: The day after the surgical procedure.

^a^ Heart rhythm disorder (1 case), veinitis or lymphangitis (4 cases), thrombophlebitis (1 cases), localized hypoesthesia under the inguinal incision (7 cases), urinary retention (5 cases), Parkinsonian decompensation (1 case), other (6 cases).

^b^ Urinary retention (2 cases).

^c^ Subcutaneous seromas or hematomas healing spontaneously (n = 42), not infected deep hematomas (n = 7).

^d^ Subcutaneous seromas (18 cases).

^e^ Deep hematoma, reintervention at D7 simple outcome.

^f^ Orchitis (1 case).

^g^ Deep hematoma requiring transfusion (1 case), hematoma re-operated on day 7 (1 case (f)), pulmonary embolism with hematoma treated as an outpatient (1 case), urinary retention managed by urologists (1 case).

### Two-Year PROM

In total, 100 of 125 (80%) SH patients and 1,470 of 1,661 (88.50%) NSH patients were reached by the clinical research assistant and answered all or almost all the questions of the formatted questionnaire (Table 4). In total, 99% of patients in each group assessed their groin to be solid. One (1%) in the SH group and 11 (0.80%) described a bulge or a tumefaction in their operated groin. Five (5%) in the SH group and 34 (2.49%) in the NSH group mentioned either moderate or severe pain. The difference was not statistically significant (p > 0.05). Similarly, the potential impact of these late symptoms (if present) on their daily life was extremely low. Only 1 (0.98%) in SH and 6 (0.44%) in NSH assessed their late symptoms as more bothersome than their preoperative symptoms. Overall, no statistically significant difference was found between the two studied groups in terms of their late PROM.

**TABLE 4:** Two-year patient related outcomes measure (PROM).

	NSH	SH	
N (%)	1,661	125	
Patients not reached/phone questionnaire (N, %)	191 (11.50)	25 (20)	P < 0.01
Q1. Since your operation does your abdominal wall seem (N answers)	1,470	100	
Solid	1,466 (99.72)	99 (99)	p > 0.05
Not solid	4	1 (1)	
Q2. Do you have a new hernia or bulge in the operated groin? (N answers)	1,363	100	
No	1,352 (99.19)	99 (99)	p > 0.05
Yes	11 (0.80)	1 (1)	
Q3. Do you currently feel any pain or local discomfort? (N answers)	1,362	100	
No (asymptomatic)	1,237 (90.82)	91 (91)	p > 0.05
Yes	125 (9.18)	9 (9)	
Mild pain or discomfort	91 (6.68)	4 (4)	
Moderate pain	28 (2.05)	5 (5)	p > 0.05
Severe pain	6(0.44)	0 (0)	
Q4. Impact of symptoms (N answers)	1,494	112	
No symptoms	1,378 (92.23)	103 (91.96)	p > 0.05
Symptoms	116 (7.76)	9 (8.03)	
Do not interfere with your daily life	105 (7.03)	8 (7.14)	
Allow to pursue the ongoing activity	6(0.40)	0 (0)	
Cause a temporary interruption of activity	2 (0.13)	1 (0.89)	
Prevent certain activities (impairment)	3(0.20)	0 (0)	
Q5. Late vs pre-operative symptoms. (N answers)	1,361	102	
No late symptoms	1,243 (91.32)	94 (92.15)	p > 0.05
Late symptoms	118(8.67)	8 (7.84)	
Less bothersome than the hernia	112 (8.23)	7 (6.86)	p > 0.05
More bothersome than the hernia	6 (0.44)	1 (0.98)	
Q6. How do you assess the result of your hernia operation (N answers)	1,352	98	
Excellent or good	1,339 (99.03)	86 (97.95	p > 0.05
Medium	10 (0.74)	1 (1.02)	
Bad	3 (0.22)	1 (1.02)	

### Identified Late Complications

At 1 year, 84 of the 125 SH patients, and 870 of the 1879 NSH patients had already completed their first annual telephone questionnaires; additionally, 29 of the 125 SH patients, and 502 of the 1879 NSH patients attended their systematically proposed clinical visits (Table 5). These combined controls allowed for the identification of the following late complications: In the SH group, only one complication (superficial infection) was recorded, which was resolved after reoperation. In the NSH group, six complications (5 reoperations) occurred in four patients, including two hernia recurrences, one superficial infection, one chronic sinus, and two mesh removals (Table 5). These late complications were rare in both studied groups, with no statistically significant difference between them.

**TABLE 5:** Identified late complications.

N *(%)*	NSH	SH	*P*. value
Patients	**1,661**	125	
Missing data	**289 *(17.39)***	12 *(9.60)*	**p < 0.01**
Patients followed	**1,372 *(82.60)***	113 *(90.40)*	**p = 0.02**
Phone questionnaire completed	**870*(52.38)***	84 *(67.2)*	**p = 0.01**
Patients attending the clinical visit	**502 *(30.22)***	29 *(23.20)*	p > 0.05
Complications/patients	**6 complications/4 patients**	1 complication/1 patient	p > 0.05
Testicular atrophy	**0**	0	
Bowel obstruction or erosion	**0**	0	
Late superficial infection operated	**1**	1	
Chronic sinus	_**1**_ ^a^	0	
Mesh removal	_**2**_ ^b^ ^,^ ^c^	0	
Recurrences	**3**	0	
Reinterventions	**5 (0.36)**	1 (0.9)	p > 0.05^d^

Percentages (in italics) were calculated on non-empty values; p values < 0.05 are in bold Chronic sinus operated twice

^a^ (Mesh removal, recurrence) Mesh removal for meshoma.

^b^ (in other center), for abscessed sigmoid diverticulosis.

^c^ (in other center).

^d^ Fischer exact test.

## Discussion

### Key Results

In the present comparative study, the first to be published on scrotal hernia repaired with the MOPP technique, the conversion rate was less than 1%, while complications (postoperative and late) and recurrence were low and similar to those observed in non-scrotal MOPP repair. Thus, this study shows taht the MOPP techniqueis feasible, safe and effective for scrotal SH S1 encountered in Europe [18, 27]. In the classification proposed by Tran et al. [18] the scrotal hernias are subdivided into S1 (upper third of the thigh), S2 (middle third of the thigh), S3 (lower third of the thigh/patella), and Sn (IR) in case of irreducibility. All scrotal hernias treated in this series were type S1, according to the previously mentioned classification. Thus, the external validity of the present study does not apply to types S2 and S3 encountered in low- or middle-income countries (LMIC). Moreover, the considerable experience in this field of our LMIC colleagues [19] may help us to figure out how to operate on the rare S3 cases we may 1 day be faced with. In the recently published “Systematic review and guidelines for the management of scrotal inguinal hernias” [18] three techniques were evaluated: the Lichtenstein technique, the totally extraperitoneal laparoscopic (TEP) repair, and the trans-abdominal laparoscopic (TAPP) repair. Due to a lack of published data, open repair other than the Lichtenstein techniques was not considered in the key questions of these guidelines. The present monocentric prospective exploratory study showed that i) the MOPP technique is feasible, safe and effective in scrotal repair for the scrotal hernias (S1) encountered in Europe, ii) the overall results of MOPP used in scrotal hernia (SH) repair were not statistically different from those of MOPP used in common groin hernia repair (NSH), iii) the conversion rate in S1 scrotal hernia repair, was 0.8% (1/126), which is very low compared to what has been published for laparoscopic techniques, especially TEP.

The conversion rate of TEP in SH repair was 25% in the 23 selected series reviewed in Tran et al. systematic review and guidelines [18]. In the series by Bansal et al. [28], TEP repair was successful in 64 patients (75.3%), converted to TAPP in 15 patients (17.6%) and to open in six patients (7.1%). TAPP repair was successful in 53 patients (89.8%) and was converted to open repair in six patients (10.2%).

In the event of technical difficulties, conversion from MOPP to Lichtenstein is easier, and quicker than from laparoscopic techniques in which a resettlement is required. Additionally, unlike African SH patients who are predominantly young, European SH patients are older and have comorbidities, as shown in the present study in which the mean age was close to 70 years, with 5 (4.5%) patients classified as ASA 3 or ASA4. In the present MOPP study, 92% of the patients received a “light” general anaesthesia with a laryngeal mask, without tracheal intubation or curarisation. The conversion rate observed in the present study was low for three main reasons: i) all the SH hernias were S1 type; ii) due to the inclusion/exclusion criteria, the cases studied were, hence, highly selected cases (Figure 1); While only one planned MOPP had to be converted to Lichtenstein, in 43 other cases Lichtenstein was our first choice. Thus, the Lichtenstein technique remains our fallback technique. Additionally, a disadvantage of the TIPP approach that is regularly cited is the need for dissection in both planes thus virtually hampering a possible approach in a “virgin” plane. In fact, this is not as significant as it appears to be. As shown in this series, the recurrences are rare after this preperitoneal open technique and can be repaired by open (because the initial superficial inguinal dissection was not extensive) or laparoscopic TAPP technique. All repairs were performed by a surgeon very experienced in this procedure.

### The Results of MOPP Were Globally the Same in SH Hernias Compared With Non-SH Hernias

The aim of the present study was not to assess the benefit/drawback balance between MOPP and other techniques in SH repair, which is the point of our following study [29] comparing head-to-head TIPP/MOPP versus Lichtenstein and TIPP/MOPP versus laparoscopic repair. Rather, the aim of this first step was to investigate whether MOPP is feasible and safe in scrotal hernias to use NSH as a control population. What we found is that, in expert hands, MOPP is feasible and effective in S1 scrotal hernias, with overall results similar to those of non-scrotal groin hernias. In particular, the low rate of identified recurrences (Table 5) the low rate of chronic postoperative inguinal pain (CPIP), both severe (0% vs. 0.44%; p > 0.05), and moderate pain (5% vs. 2.05%), p > 0.05 (Table 4). Some differences remain to be underlined: In terms of pain/discomfort/Q.O.L. SH patients benefitted the most from their surgery (high improvements) with, in addition, an extremely low rate of late complications (Table 5). The preoperative pain/discomfort and the Q.O.L alterations were significantly more important in the SH patients (Table 1), while their postoperative pain and PROM (Table 4) were low and similar to those of the NSH patients. On the other hand, the rate of postoperative SSO on day 30 (Table 3) was significantly higher in the SH group than in the NSH group (14.40% vs. 2.98%; p < 0.0001). These surgical site occurrences (SSO) consisted only of non-infected seromas. No early periprosthetic infection occurred in either group. All day 30 postoperative complications in the SH group were classified as Clavien I/II, none as Clavien III or higher. Similar findings were reported in the Herniamed registry [27], in which scrotal hernias demonstrated an unfavourable association with postoperative complication rates but a favourable association with chronic pain rates. In both groups, probably due to the minimally invasive nature of the MOPP technique, general complications were rare and benign (Table 3). Thus the longer hospital stay in SH patients was probably related to their higher rate of SSO and to intraoperative difficulties. The operating time was longer (58 min vs. 39 min; p < 0.0001) in the SH group, due to technical difficulties and modifications to the standard MOPP technique.

### Technical Modifications to the Standard MOPP Technique Required for S1 Hernia Repair

It is advisable not to dissect the sac too far distally and therefore to leave its bottom after having opened it widely. An increased risk of seroma is preferable to an increased risk of testicular ischaemia and haematoma [18]. As much as possible, it is preferable to implant a large mesh that broadly covers the entire Fruchaud’s myo-pectineal area. A memory ring or a peripheral reinforcement of the mesh, greatly helps the deployment of the mesh. In the present series, a large mesh was implanted significantly more often in SH than in NSH patients (83.20% vs. 65.31%; p < 0.0001). In NSH patients, mesh fixation was rarely used (Table 2). In large defects, especially medial ones, using a suture to fix the prosthesis to the Cooper’s ligament is recommended by the guidelines [18, 27]. In series reported in the Herniasurge guidelines [27] scrotal hernias are largely drained. Similarly, in the systematic review by Tran et al. [18], some articles [30] suggest that drainage may reduce the occurrence of either haematomas or seromas. In the present monocentric experience, the surgeon never used a drain even in the repair of the largest S1 hernias. While 18 cases (14.40%) of seromas or small haematomas occurred, they never required specific treatment and gradually resolved without significant patient discomfort.

### Limitations

This study has several limitations. This is a non-randomised comparative study but it is based on monocentric exhaustive prospectively collected data in a national registry. The two groups may appear poorly comparable (Table 1), suggesting the need for propensity score matching. In fact, due to the large number of patients, small differences may be statistically significant while being clinically poorly significant. This is the case in our two populations: Mean age (69.08 vs. 68.86), mean BMI (24.62 vs. 24.93), frequency of patients on anticoagulant therapy (14.08% vs. 12.80%), active smokers (19.56% vs. 20.80%), ASA 1–2 (96.76% vs. 95.49%). Preoperative pain (and discomfort) was found to be higher in scrotal hernias than in non-scrotal ones. This is well-known and the subject of many studies and is inherent to the scrotal nature of the hernia. This is a monocentric series, from one surgeon who is highly skilled in this technique, which limits the external validity of the study. Regular follow-up was mainly achieved by telephone questionnaire and not all the patients had a late clinical visit. Thus, small sub-clinical recurrences may have been missed. However, the methodology was the same in the two studied groups. While a telephone questionnaire is not the best tool for detecting small asymptomatic recurrences, even with the PINQ-Phone manoeuvre [24], it is a reliable tool to detect rehospitalisation (in the same or another hospital), reoperation and its causes, late infections, late mesh removals, and other late complications such as bowel obstructions (all events not ignored by the patients). And an excellent tool to assess PROM, Q.O.L and CPIP [31].

### Strengths

On the other hand, this study has several strengths. This is a monocentric, single-operator (homogeneous) study based on an exhaustive registration of cases and a high follow-up rate. Almost 90% (SH) and 83% (NSH) of the patients were followed up for more than 1 year, either by a telephone questionnaire conducted by a specialised clinical research assistant, independent from the surgical team or by clinical visits to the surgeon’s office, which patients were systematically encouraged to attend.

## Conclusion

The present study clearly demonstrated the feasibility and the safety of the MOPP technique in S1 scrotal hernia repairs. The results of this first step study led us to set up a complementary study in scrotal hernia repairs, comparing head-to-head the results of TIPP/MOPP versus Lichtenstein technique and TIPP/MOPP versus laparoscopic techniques.

## Data Availability

The original contributions presented in the study are included in the article/supplementary material, further inquiries can be directed to the corresponding author.
